# Letter to the editor regarding “Intracranial hemorrhage after ischemic stroke in patients on direct oral anticoagulants: results from a prospective observational study” by Purrucker et al

**DOI:** 10.1186/s42466-026-00484-6

**Published:** 2026-06-02

**Authors:** Matija Zupan, Mišo Šabovič, Senta Frol

**Affiliations:** 1 Department of Vascular Neurology, University Medical Centre Ljubljana, Ljubljana, Slovenia; 2 Faculty of Medicine, University of Ljubljana, Ljubljana, Slovenia; 3 Department of Vascular Disorders, University Medical Center Ljubljana, Ljubljana, Slovenia; 4 Department of Vascular Neurology, Faculty of Medicine, Ljubljana University, Zaloška 2, Ljubljana, 1000 Slovenia

**Keywords:** Acute ischemic stroke, Direct oral anticoagulants, Intravenous thrombolysis

Dear Editor,

We read with great interest the prospective multicenter study by Purrucker et al. reporting intracranial hemorrhage risk after acute ischemic stroke (AIS) in patients receiving oral anticoagulation within the RASUNOA-Prime registry [1].

The authors address a clinically highly relevant and increasingly frequent scenario in modern stroke care, where approximately 10% of AIS patients are taking an oral anticoagulant at the time of presentation [2], and should be commended for providing prospective data in an area that has long relied predominantly on retrospective analyses.

A major strength of this study lies in its prospective design, predefined recruitment strategy, and centralized blinded neuroimaging assessment. These methodological features substantially strengthen the level of evidence compared with prior observational reports. Importantly, the authors demonstrate low rates of symptomatic intracranial hemorrhage and comparable bleeding risk across anticoagulation groups, including patients treated with direct oral anticoagulants (DOACs), even after adjustment for thrombolytic therapy. Another important finding of the present study is lower stroke severity in DOAC-treated patients, which is biologically plausible given a more consistent anticoagulation efficacy, with consequently smaller infarct volumes, and reduced size and stability of thrombi under DOAC treatment [3].

These findings add meaningful reassurance regarding the safety profile of reperfusion therapies in anticoagulated patients. Accumulating data increasingly challenge the traditional perception of recent DOAC intake as an almost absolute contraindication to intravenous thrombolysis (IVT). While earlier evidence was largely retrospective, prospective studies such as RASUNOA-Prime mark an important evolution of the field. Together with ongoing prospective trials, including the anticipated results of the DO-IT study [4], the evidence base is steadily moving toward a more nuanced understanding of sICH risk in this population. We acknowledge, however, that selected patient populations in prospective registries may not fully capture the higher-risk scenarios that clinicians may encounter in routine emergency care.

Despite this progress, many international recommendations and local protocols continue to regard DOAC therapy as a near-categorical exclusion criterion for IVT [5]. This discrepancy between evolving evidence and clinical guidance may lead to systematic undertreatment of patients with AIS. We believe that the stroke community should begin reconsidering whether expert consensus statements and local treatment algorithms ought to gradually adapt to contemporary data, shifting from rigid contraindications toward individualized decision-making integrating clinical context and available laboratory information.

An additional question emerging from current clinical practice concerns reversal strategies prior to IVT, particularly the administration of idarucizumab in patients receiving dabigatran [5]. Reversal is frequently performed for dabigatran but has no equivalent strategy for factor Xa inhibitors, creating both mechanistic and practical inconsistencies. Beyond economic considerations, the biological implications of immediate anticoagulation reversal remain incompletely understood. Emerging data suggest low hemorrhagic complication rates even without systematic reversal, raising questions about the incremental biological and logistical benefit of antidote administration, and supporting the need to critically reassess the routine use of pharmacological reversal.

The urgency of addressing these issues is underscored by rapidly changing epidemiology. The number of patients receiving DOAC therapy continues to rise due to increasing prevalence of atrial fibrillation, expanding indications for oral anticoagulation, and demographic aging. Consequently, stroke physicians are encountering a growing population of patients presenting with AIS while receiving DOAC treatment. Developing pragmatic, evidence-based strategies is therefore a clear priority. The discussion is gradually moving beyond whether change is needed toward how it can be responsibly implemented, signaling that the field may be approaching a paradigm shift in the management of anticoagulated stroke patients.

In conclusion, the work by Purrucker et al. [1] represents an important step toward this goal by providing reassuring prospective data from routine clinical practice. At the same time, it highlights the need for intensified international collaboration to generate harmonized expert recommendations while awaiting results from ongoing prospective trials. In this evolving context, continued reliance on automatic exclusion policies appears increasingly misaligned with the contemporary balance between hemorrhagic risk and potential functional benefit.

## Data Availability

The dataset is available on request from the authors.
